# A novel polyamine blockade therapy activates an anti-tumor immune response

**DOI:** 10.18632/oncotarget.20493

**Published:** 2017-08-24

**Authors:** Eric T. Alexander, Allyson Minton, Molly C. Peters, Otto Phanstiel, Susan K. Gilmour

**Affiliations:** ^1^ Lankenau Institute for Medical Research, Wynnewood, PA 19096, USA; ^2^ University of Central Florida, Biomolecular Research Annex, Orlando, FL 32826-3227, USA

**Keywords:** polyamines, difluoromethylornithine, transport inhibitor, immunomodulation, tumor microenvironment

## Abstract

Most tumors maintain elevated levels of polyamines to support their growth and survival. This study explores the anti-tumor effect of polyamine starvation via both inhibiting polyamine biosynthesis and blocking the upregulated import of polyamines into the tumor. We demonstrate that polyamine blockade therapy (PBT) co-treatment with both DFMO and a novel polyamine transport inhibitor, Trimer PTI, significantly inhibits tumor growth more than treatment with DFMO or the Trimer PTI alone. The anti-tumor effect of PBT was lost in mice where CD4^+^ and CD8^+^ T cells were antibody depleted, implying that PBT stimulates an anti-tumor immune effect that is T-cell dependent. The PBT anti-tumor effect was accompanied by an increase in granzyme B^+^, IFN-γ^+^ CD8^+^ T-cells and a decrease in immunosuppressive tumor infiltrating cells including Gr-1^+^CD11b^+^ myeloid derived suppressor cells (MDSCs), CD4^+^CD25^+^ Tregs, and CD206^+^F4/80^+^ M2 macrophages. Stimulation with tumor-specific peptides elicited elevated antigen-specific IFN-γ secretion in splenocytes from PBT-treated mice, indicating that PBT treatment stimulates the activation of T-cells in a tumor-specific manner. These data show that combined treatment with both DFMO and the Trimer PTI not only deprives polyamine-addicted tumor cells of polyamines, but also relieves polyamine-mediated immunosuppression in the tumor microenvironment, thus allowing the activation of tumoricidal T-cells.

## INTRODUCTION

Cancer cells develop many diverse and complex mechanisms to evade the effects of chemotherapeutics, particularly drugs that target a specific signaling pathway [1]. This chemoresistance remains a significant obstacle to successful cancer therapy. Given the heterogeneity and plastic nature of most tumors, a better approach may be to target tumor modifiers that support multiple components of the tumor including both tumor epithelial cells as well as stromal and infiltrating immune cells in the tumor microenvironment [2–4]. Compared to normal cells, tumor cells have been shown to contain elevated levels of polyamines (putrescine, spermidine, and spermine) [5–8]. Polyamines are amino acid-derived polycations that have been implicated in a wide array of biological processes, and they are essential for cellular proliferation, differentiation, and cell death [8–16]. Intracellular polyamine levels are maintained via tightly-regulated biosynthetic, catabolic, and uptake and export pathways [13]. Oncogenes such as MYC and RAS both upregulate polyamine biosynthesis [17–19] and increase cellular uptake of polyamines by inducing the polyamine transport system (PTS) [20, 21]. In order to meet their huge metabolic needs, most tumors have a greatly increased need for polyamines compared to normal cells and, consequently, polyamines are potent modifiers of tumor development [22].

Targeting polyamine metabolism has long been an attractive approach to cancer chemotherapy. Ornithine decarboxylase (ODC), the rate-limiting enzyme in polyamine biosynthesis, is elevated in tumors and is an early marker and promoter of tumorigenesis [8, 23–26]. Although α-difluoromethylornithine (DFMO), an irreversible inhibitor of ODC activity, showed promise as a chemotherapeutic agent *in vitro*, it has had only moderate success in treating cancer patients [8]. Subsequent studies discovered that its effect on inhibiting polyamine biosynthesis was abrogated *in vivo* with a compensatory increased activity of the PTS in tumor cells with resulting increased uptake of polyamines derived from the diet and gut flora into the tumor cells [22, 27]. Thus, to polyamine-starve a tumor, both polyamine biosynthesis as well as polyamine transport must be inhibited. Following the discovery that DFMO treatment upregulates the PTS in tumors, work has focused on finding drugs that can target the PTS.

To starve tumor cells of polyamines that are essential for their growth and survival, we have developed a new polyamine blockade therapy (PBT) that includes *a combination of* 1) DFMO and 2) a novel polyamine transport inhibitor (PTI). This approach exploits the oncogene and DFMO-induced PTS activity in tumor cells by inhibiting the PTS with a novel Trimer PTI [28, 29]. Both natural polyamines and polyamine-based drugs are imported into tumors via this specific polyamine uptake system. In contrast, normal cells are predicted to be significantly less sensitive to the Trimer PTI due to their low PTS activity [30]. In this report, we reveal for the first time the anti-tumor efficacy of combination treatment with DFMO and the Trimer PTI in two animal tumor models. We show that this polyamine-targeted therapy provides a dual attack on tumors by starving the tumors of the polyamine growth factors needed for their proliferation and survival and by activating an immune attack on these tumors.

## RESULTS

### PBT therapy reduces tumor growth and progression

**Figure 1:**
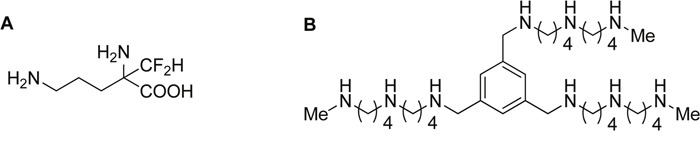
Structure of DFMO and Trimer PTI Polyamine blockade therapy consists of combined treatment with α-difluoromethylornithine (DFMO, an ODC inhibitor), and the Trimer PTI (N1, N1′, N1″-(benzene-1, 3, 5-triyltris(methylene))tris(N4-(4-(methylamino)butyl)butane- 1, 4-diamine), an inhibitor of the polyamine transport system [29].

**Figure 2:**
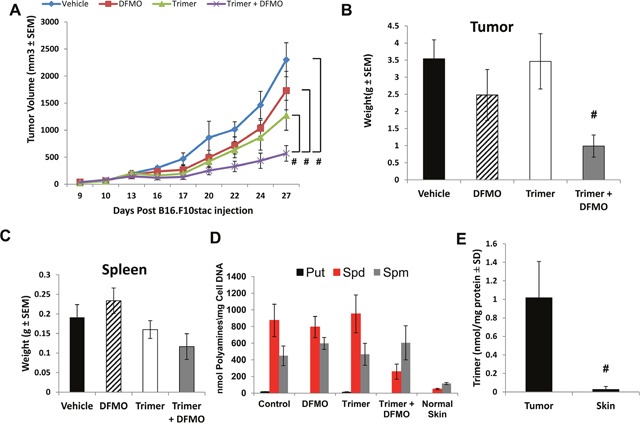
B16F10-sTAC tumor growth inhibition with DFMO and Trimer PTI **(A)** Mice were subcutaneously injected with 5×10^5^ B16F10-sTAC melanoma cells. When tumors were 50-100 mm^3^ in size, treatment was initiated with either saline, 0.25% DFMO (w/v) in the drinking water, Trimer PTI (i.p., 3 mg/kg, once a day) or both DFMO and Trimer PTI. Graph shows B16F10-sTAC tumor growth under different treatments (mean tumor volume ± SEM). **(B)** Spleen weight was determined upon sacrifice (mean ± SEM). **(C)** Upon sacrifice, tumors were excised and weighed (mean ± SEM). **(D)** Polyamine levels were determined in tumors by HPLC and normalized to DNA levels in the tissue extracts (nmol/mg DNA). **(E)** Tumor and non-tumor bearing skin tissues were excised from PBT-treated mice, flash frozen, finely pulverized in liquid nitrogen with mortar and pestle, homogenized in a solution of 33% water, 66% methanol and 1% acetic acid, and then centrifuged at 5000 rpm for 10 min at 25°C. Trimer PTI levels were determined in tumor and skin supernatants by mass spectrometry and normalized to tissue protein concentration (nmol/mg protein). n = 5-10 mice per group; ^*^ = p ≤ 0.05 and # = p ≤ 0.01 compared to vehicle-treated mice.

To evaluate the effect of polyamine blockade therapy (PBT) we used the B16F10-sTAC melanoma model. Following subcutaneous injection of B16F10-sTAC cells expressing SIINFEKL peptide in C57/Bl6 mice, treatment was initiated when tumors were between 50-100 mm^3^ in size. Mice were administered Trimer PTI (i.p. injection 3 mg/kg daily), with or without 0.25% DFMO (w/v) in the drinking water (Figure 1). Treatment with either DFMO or Trimer PTI individually only modestly reduced B16F10-sTAC tumor growth compared to vehicle treatment (Figure 2A and 2B). However, there was a significant inhibitory effect on tumor growth in mice treated with both DFMO and the Trimer PTI with a 4-fold reduction in final tumor weight compared to vehicle treated mice. Treatment with Trimer PTI with or without DFMO had no significant effect on spleen weight (Figure 2C). High-performance liquid chromatography (HPLC) analysis of the polyamine content in tumors showed that all polyamines, including putrescine, spermidine, and spermine, were elevated in the tumors compared to non-tumor bearing skin. Treatment with DFMO alone reduced the levels of putrescine compared to vehicle-treated mice, whereas treatment with Trimer PTI alone had no discernible effect on polyamine levels (Figure 2D). However, co-treatment with both DFMO and the Trimer PTI significantly reduced the levels of both putrescine and spermidine in the tumors compared with vehicle treated mice (Figure 2D). Mass spectrometry analysis demonstrated that the Trimer PTI accumulated preferentially in the tumor (30 fold) of PBT- treated mice compared to levels detected in surrounding non-tumor bearing skin (Figure 2E).

**Figure 3:**
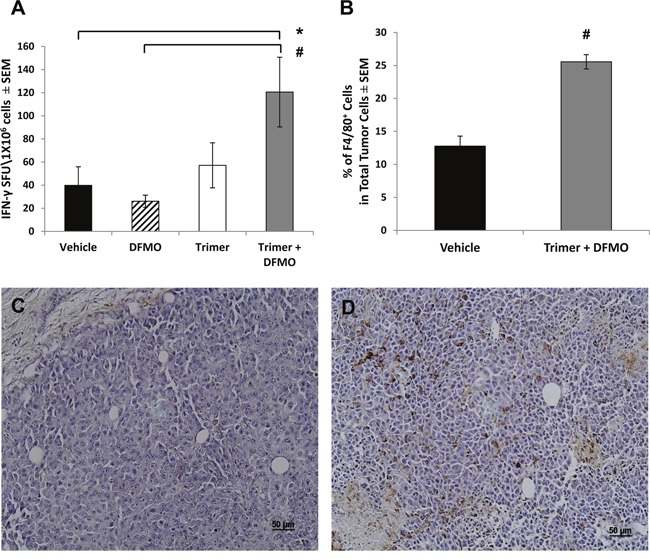
DFMO and Trimer PTI co-treatment increases cytotoxic T-cell activity and promotes macrophage infiltration of the tumor **(A)** The frequency of IFN-γ producing T-cells was measured by the ELISpot assay as IFN-γ spot forming units (SFU) per million spleen cells. **(B)** The number of F4/80^+^ cells per million B16F10-sTAC melanoma cells from either vehicle treated mice or mice co-treated with DFMO and Trimer PTI. Representative images of B16F10-sTAC tumor sections from mice treated with vehicle **(C)** or DFMO and Trimer PTI **(D)** and stained for F4/80^+^ macrophages. n = 5-10 mice per group; ^*^ = p ≤ 0.05 and # = p ≤ 0.01 compared to vehicle treated mice.

**Figure 4:**
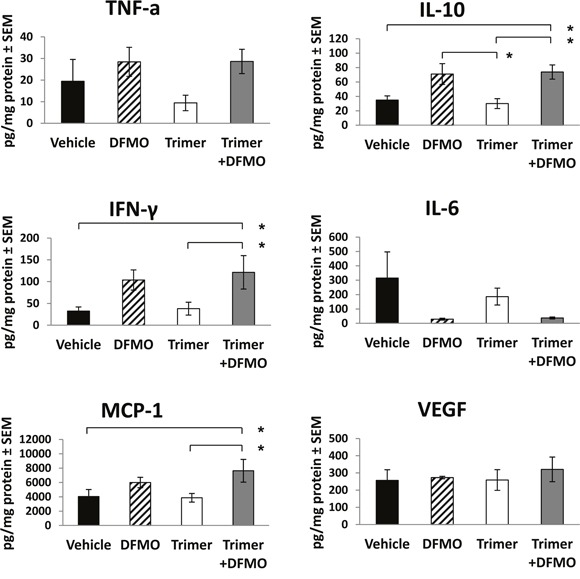
DFMO and Trimer PTI co-treatment increases levels of pro-inflammatory cytokines in B16F10-sTAC tumors Upon sacrifice, tumors were excised, flash frozen in liquid nitrogen and then homogenized to produce tumor lysates which were assayed for levels of TNF-α, IL-10, IFN-γ, IL-6, MCP-1 and VEGF by Cytokine Bead Array or ELISA. n = 5-10 mice per group; ^*^ = p ≤ 0.05 and # = p ≤ 0.01 compared to indicated group.

The decreased tumor growth in mice treated with DFMO and Trimer PTI was associated with a significant increase in the number of IFN-γ producing splenocytes as measured by the ELISpot assay following *ex vivo* stimulation with SIINFEKL peptide (Figure 3A) as well as an increase in the number of F4/80 positive macrophages in the tumor (Figure 3B). The increase in F4/80 positive macrophages in the tumors of mice co-treated with DFMO and Trimer PTI was also associated with movement of the macrophages from the periphery of the tumor, as seen in vehicle treated tumors to the interior (Figure 3C and 3D). The increased tumor infiltration of macrophages correlated with increased levels of proinflammatory cytokines including monocyte chemoattractant protein-1 (MCP-1) which is one of the key chemokines that regulate migration and infiltration of monocytes/macrophages. Treatment with DFMO or Trimer PTI individually did not significantly alter cytokine levels compared to vehicle treated mice (Figure 4). However, co-treatment with both DFMO and Trimer PTI significantly increased the levels of IL-10, IFN-γ, and MCP-1, i.e., cytokines associated with increased immune activity in the tumor and tumor microenvironment.

### Anti-tumor effects of PBT rely on host immune processes

**Figure 5:**
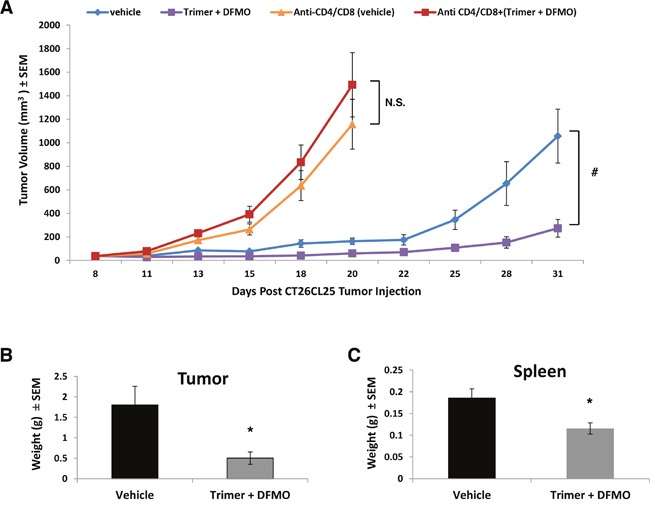
Depletion of CD4^+^ and CD8^+^ T-cells reverses PBT inhibition of tumor growth **(A)** Mice were subcutaneously injected with 5×10^5^ CT26.CL25 colon carcinoma cells. When tumors were 50-100 mm^3^ in size, treatment was initiated with either saline or 0.25% DFMO (w/v) in the drinking water plus Trimer PTI (i.p. 3 mg/kg, once a day). Mice in the anti-CD4/CD8 groups were i.p. injected with 75 μg of anti-CD4 and anti-CD8 antibodies every three days starting 3 days prior to the initiation of treatment with a total of four doses. Graph shows CT26.CL25 tumor growth under different treatments (mean tumor volume ± SEM). Upon sacrifice, tumors **(B)** and spleens **(C)** were excised and weighed (mean ± SEM). n = 5-10 mice per group; ^*^ = p ≤ 0.05 and # = p ≤ 0.01 compared to indicated group.

Because decreased tumor growth in mice co-treated with DFMO and Trimer PTI was associated with increased T-cell IFN-γ production as measured by ELISpot assay as well as increased levels of IL-10, MCP-1 and IFN-γ, we hypothesized that PBT anti-tumorigenic properties may be dependent on host immune system competence. To test this, mice with subcutaneous CT26.CL25 colon carcinoma tumors were treated with anti-CD4 and anti-CD8 antibodies to deplete CD4^+^ and CD8^+^ T-cells before and during the treatment with PBT. As expected, tumors in mice treated with anti-CD4/CD8 antibodies grew at an accelerated rate compared to vehicle treated mice (Figure 5A). Whereas PBT treatment significantly reduced CT26.CL25 tumor growth in mice possessing a full complement of CD4^+^ and CD8^+^ T-cells (Figure 5A and 5B), PBT treatment had no significant inhibitory effects on tumor growth in mice that had received anti-CD4/CD8 antibodies (Figure 5A). The spleen weight of CT26.CL25 tumor bearing mice was significantly higher compared to those on PBT treatment and mice without tumors (Figure 5C).

### PBT treatment reduces specific subpopulations of suppressor and pro-tumorigenic cells while increasing T effector cell interferon-γ and granzyme B production

**Figure 6:**
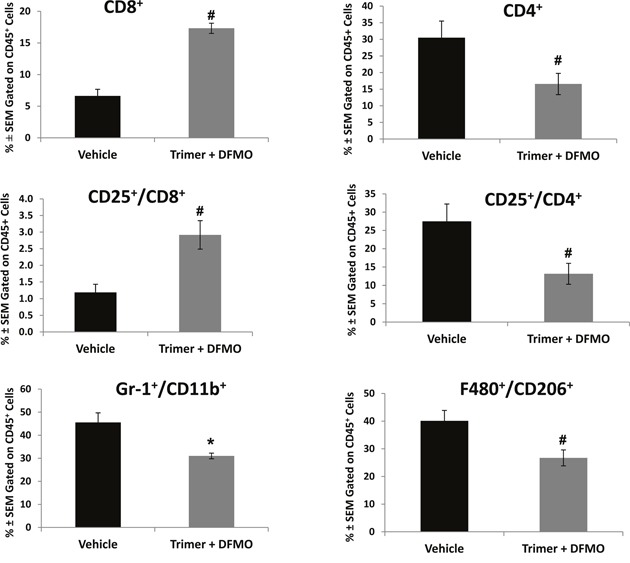
DFMO and Trimer PTI co-treatment reduces the immunosuppressive and pro-tumorigenic cells populations Upon sacrifice, tumors were excised from CT26.CL25-tumor bearing mice and processed for analysis by flow cytometry. CD45^+^ tumor cells were analyzed for the percentage of the indicated cell subpopulations. n = 5-10 mice per group; ^*^ = p ≤ 0.05 and # = p ≤ 0.01 compared to indicated group.

**Figure 7:**
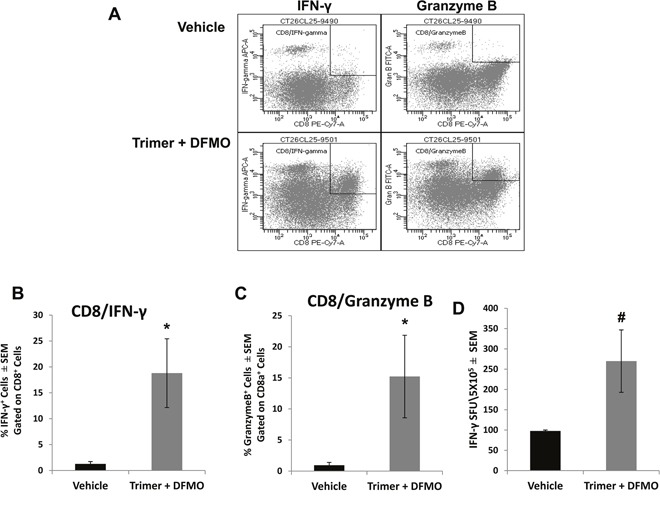
Co-treatment with DFMO and Trimer PTI increases the cytotoxic T-cell activity in the tumor Upon sacrifice, tumors were excised from CT26.CL25-tumor bearing mice and infiltrating leukocytes were isolated by discontinuous Percoll gradients. CD8^+^ T-cells were analyzed for the percentage of IFN-γ^+^ **(A** and **B)** and granzyme B^+^ **(A** and **C)** cells by flow cytometry. **(D)** The frequency of IFN-γ producing T-cells was measured by the ELISpot assay as IFN-γ spot forming units (SFU) per 5 x10^5^ tumor infiltrating leukocytes. n = 5-10 mice per group; ^*^ = p ≤ 0.05 and # = p ≤ 0.01 compared to indicated group.

Subpopulations of immune cell infiltrates in CT26.CL25 tumors were analyzed by flow cytometry following isolation of tumor leukocytes using a discontinuous Percoll gradient. Treatment with PBT significantly decreased the populations of Gr1^+^CD11b^+^ myeloid derived suppresser cells (MDSC) and CD25^+^CD4^+^ T regulatory cells (Tregs), major immunosuppressive cell types found in many different tumor types (Figure 6). PBT treatment also significantly reduced the levels of F4/80^+^CD206^+^ M2 macrophages that have been shown to support and promote tumor growth in a broad range of tumors. Similar changes were also noted in the FACS analyses of splenocytes from vehicle and PBT treated mice. Treatment with PBT significantly reduced M2 macrophages (F4/80^+^CD206^+^), MDSCs (arginase^+^/Gr1^+^/CD11b^+^) and Tregs (CD4^+^CD25^+^) populations as well as overall arginase^+^ CD45^+^ leukocytes (Supplementary Figure 1). While PBT treatment reduced the levels of immunosuppressive and pro-tumorigenic leukocytes that infiltrated the tumors, it also significantly increased the percentage of CD8^+^ cytotoxic T-cells in the tumors (Figure 6) which are immune cells responsible for directly killing tumors cells. To further characterize CD8^+^ T effector cells infiltrating the tumors, we analyzed the intracellular levels of IFN-γ and granzyme B, proteins that are released from cytotoxic T-cells to stimulate the immune system and to actively induce apoptosis in target cells, respectively. Compared to vehicle-treated mice, treatment with PBT significantly increased the percentage of CD8^+^ T-cells producing IFN-γ from 1.3 ± 0.3% to 18.8 ± 6.6% (Figure 7A and 7B), while increasing the percentage of CD8^+^ T-cells producing granzyme B to 15.2 ± 5.4% from 1.0 ± 0.3% for vehicle treated mice (Figure 7A and 7C). Isolated infiltrating leukocytes from the tumors were also assayed in an IFN-γ ELISpot assay. Treatment with PBT was associated with a significant increase in the number of IFN-γ producing isolated infiltrating leukocytes following *ex vivo* stimulation with the CT26.CL25 tumor-specific LacZ peptide (Figure 7D).

**Figure 8:**
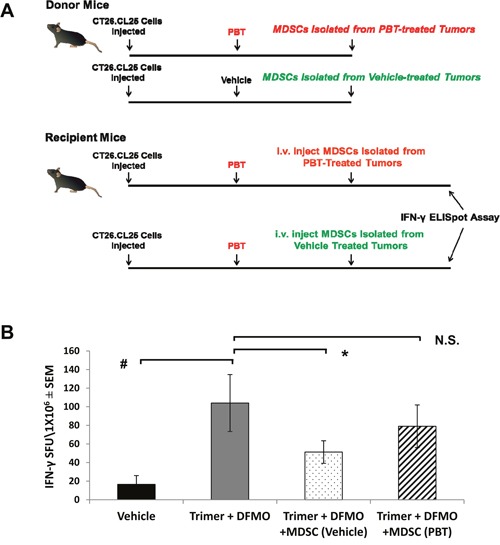
Co-treatment with DFMO and Trimer PTI decreases the immunosuppressive activity of MDSCs **(A)** One week prior to sacrifice, MDSCs were isolated from the spleens of CT26.CL25 tumor-bearing mice that were treated either with vehicle or Co-treatment with DFMO and Trimer PTI decreases. Isolated MDSCs (5.2 × 10^6^ per mouse) were then adoptively transferred into separate groups of recipient tumor-bearing mice in the PBT-treated group. **(B)** Upon sacrifice, splenocytes from the recipient mice were used to measure the frequency of IFN-γ producing T-cells by ELISpot assay following challenge with tumor-expressing β-galactosidase peptide. n = 5-10 mice per group; ^*^ = p ≤ 0.05 and # = p ≤ 0.01 compared to indicated group.

Because treatment with PBT significantly reduced MDSCs and Tregs, while increasing cytotoxic T-cell activity, we hypothesized that PBT treatment was directly effecting suppressor cell populations, which subsequently lessened suppression of CD8^+^ T-cells and increased T-cell activity (as seen by the IFN-γ ELISpot assays). To test this hypothesis, MDSCs were isolated from CT26.CL25 tumor bearing mice that were vehicle control treated or treated with PBT, and then MDSCs were adoptively transferred to recipient CT26.CL25 tumor-bearing mice treated with PBT, one week before they were sacrificed (Figure 8A). The single adoptive transfer of MDSCs from vehicle treated mice into recipient tumor-bearing mice treated with PBT blunted the PBT-induced increase in antigen-specific T-cell IFN-γ response (Figure 8B). However, the adoptive transfer of MDSCs isolated from tumor-bearing, PBT-treated mice into recipient tumor-bearing mice treated with PBT resulted in no significant reduction in tumor-specific T-cell IFN-γ production compared to that seen with PBT treated mice that did not receive a MDSC adoptive transfer. These data suggest that PBT treatment had modified or altered the MDSCs making them less immunosuppressive. Overall these results suggest that co-treatment with DFMO and Trimer PTI significantly inhibits tumor growth in multiple tumor models by reversing the immunosuppressive tumor microenvironment.

## DISCUSSION

An increased need for polyamines is essential for oncogenic activity in tumors, both in providing for biomass via dramatic increases in protein translation and in contributing to survival pathways for the tumor cells. Elevated intracellular polyamine levels in tumors are achieved by induction of polyamine biosynthesis enzymes and import pathways. The development of polyamine transport inhibitors is important since many tumor types upregulate PTS activity in the presence of polyamine biosynthesis inhibitors such as DFMO. We show here that combination PBT treatment with DFMO and the novel Trimer PTI significantly lowers intracellular tumor polyamine levels resulting in reduced tumor growth in animals. Importantly, our results demonstrate that PBT is an anti-metabolic treatment that reverts tumor-induced immunosuppression by re-conditioning the tumor microenvironment. Indeed, PBT reduction of tumor myeloid suppressor cell populations and activation of antitumor immune responses is critical for its anti-tumor efficacy since it fails to decrease tumor growth in mice lacking T cells.

Polyamines are important modulators of the immune response, particularly in the tumor microenvironment where they are found in high concentrations. Spermine inhibits the production of IL-12, TNF-α, IL-1, IL-6, MIP-1a, and MIP-1b by *in vitro*-stimulated macrophages [31, 32] and protects against lethal sepsis by attenuating local and systemic inflammatory response [33]. *In vitro* studies have also shown that polyamines suppress lymphocyte proliferation, decrease macrophage tumoricidal activity and neutrophil motility, and decrease IL-12-dependent NK cell activity [34–38]. We observed significantly elevated IL-10 levels in tumors of PBT treated mice. IL-10 is a highly pleiotropic cytokine that has been characterized as both a tumor promoter and an inhibitor of tumor progression depending on context. Multiple studies have found a positive correlation between IL-10 levels and poor prognosis in melanoma, likely due to immunosuppressive properties of IL-10 [39]. However others have found that IL-10 has potent anti-tumor effects. Emmerich *et.al*. found that intravenous administration of IL-10 resulted in the rejection of implanted tumors due to activation and expansion of resident CD8^+^ T-cells [40]. This study also reported an increase in IFN-γ and granzyme B expression and a three-fold increase in tumor-infiltrating cytotoxic leukocytes in mice receiving IL-10 [40]. We found that treatment with DFMO alone or Trimer alone was not sufficient to significantly inhibit tumor growth. However, blocking both polyamine biosynthesis and polyamine cellular uptake with PBT not only decreased tumor polyamine levels resulting in significantly reduced tumor growth but also enhanced an anti-tumor immune response. In mice with either B16F10 or CT26.CL25 tumors, splenocytes from PBT-treated mice demonstrated a significant increase in tumor-specific IFN-γ expression that was not present in tumor-bearing mice treated with DFMO or Trimer alone. Our data shows that PBT stimulates an anti-tumor immune effect that is T-cell dependent since the anti-tumor effect of PBT was lost in mice where CD4^+^ and CD8^+^ T cells were antibody-depleted. PBT anti-tumor activity was accompanied by an increase in activated CD8^+^ T-cells and a decrease in immunosuppressive tumor infiltrating cells including Gr-1^+^CD11b^+^ myeloid derived suppressor cells (MDSCs), CD4^+^CD25^+^ Tregs, and CD206^+^F4/80^+^ M2 macrophages. It is important to note that neither DFMO nor Trimer PTI are cytotoxic drugs, but the anti-tumor activity of PBT combination treatment deprives polyamine-addicted tumors of polyamines that they need for survival and also relieves polyamine-mediated immunosuppression in the tumor microenvironment.

The Trimer PTI is a competitive inhibitor of the PTS that out-competes native polyamines for binding to the cell surface receptors (e.g. heparan sulfate proteoglycans) of the PTS [30, 41]. Normal cells can synthesize sufficient levels of polyamines and have very low PTS activity compared to tumor cells that have a much greater need for polyamines. Mass spec analysis shows that the high PTS activity in tumor cells results in higher accumulation of Trimer PTI in tumors compared to normal tissue, thus reducing toxicity in non-tumor tissues while more specifically reducing polyamine levels in the tumor. Another polyamine-based PTI, AMXT1501, has also been shown to have anti-tumor efficacy when combined with DFMO [42]. However, the design of the lipophilic linear palmitic acid-lysine-spermine structure of AMXT1501 differs from the 1, 3, 5-trisubstituted benzene of the Trimer PTI [29]. For example, the Trimer PTI is more water-soluble, is orally available, and presents three homospermidine ‘messages’ to the receptor compared to the N^1^-acylated spermine motif presented by AMXT1501 [29]. Since the N^1^-position in AMXT1501 is N-acylated, this design converts the spermine headgroup into a modified N-alkylated spermidine motif. We note that the homospermidine motif in the Trimer PTI has been shown *in vitro* to outperform the spermidine tail available in AMXT1501 in terms of selectively targeting cells with active polyamine transport [29, 43, 44]. While both PTI approaches have their merits, we noted that the *N*-methylation of the terminal primary amines of the Trimer PTI provides additional metabolic stability to amine oxidases [45], and blood and tissue analyses have revealed that its metabolism via demethylation creates an even more potent PTI [29]. In summary, the Trimer PTI possesses a balance of properties that include low toxicity, high potency, improved targeting, and metabolic stability.

PBT-activation of a tumor immune response may be due to a direct activating effect on T-cell tumor infiltrates as well as indirect inhibitory effects on myeloid cell subpopulations that suppress T-cell activation. A variety of tumor-infiltrating immunosuppressive myeloid populations, including MDSCs, M2 macrophages, and Treg populations, have been shown to suppress cytotoxic T-cell activity. Our data show that PBT decreases levels of multiple immunosuppressive myeloid cell populations that infiltrate tumors. In contrast, another study reported that treatment with higher doses of DFMO alone did not decrease MDSC accumulation in tumor-bearing mice even though DFMO-treatment of MDSCs in short-term culture inhibited their suppression of T-cell proliferation in an arginase-dependent manner [46]. Thus, it appears that polyamine depletion with both DFMO and the Trimer PTI is necessary to decrease MDSC accumulation in tumor-bearing animals. This is further reinforced with our experimental data showing that adoptive transfer of MDSCs reduced the tumor-specific T-cell IFN-γ production that was stimulated in PBT-treated tumor-bearing mice. However, adoptive transfer of MDSCs from PBT-treated mice did not significantly reduce this tumor-specific T-cell IFN-γ production, suggesting that blocking both polyamine biosynthesis and transport is necessary *in vivo* to impair the accumulation and immunosuppressive function of MDSCs. Although the molecular basis for PBT-mediated inhibitory effects on myeloid immunosuppressive activity remains to be determined, previous studies have shown that DFMO blocks IL-4 induction of arginase activity in macrophages [42] as well as impairing the immunosuppressive activity of MDSCs via reducing their arginase activity [46]. PBT reduces arginase activity that is induced in not only immunosuppressive myeloid cells but also in tumor epithelial cells with elevated polyamine levels [42]. Furthermore, a recent study has shown that polyamines released by MDSCs can confer an indoleamine 2, 3-dioxygenase 1 (IDO1)-dependent, immunosuppressive phenotype on dendritic cells [47]. Thus, it is likely that PBT activates an anti-tumor immune response via multiple mechanisms affecting the metabolism of both tumor epithelial cells as well as immunosuppressive tumor-associated cell populations.

These data show that combined treatment with both DFMO and the Trimer PTI not only deprives polyamine-addicted tumor cells of polyamines, but also relieves polyamine-mediated immunosuppression in the tumor microenvironment, thus allowing the activation of tumoricidal T-cells. Tumor synthesis and release of polyamines contributes to immune editing of tumors and the selection of immunosuppressive cells in the tumor microenvironment. This polyamine blocking therapy offers exciting potential as adjunct cancer treatment both with conventional chemotherapeutic agents and in stimulating anti-tumor immune responses with tumor immunotherapies.

## MATERIALS AND METHODS

### Animals

Female C57Bl6 or Balb/C mice were obtained from Charles Rivers/NCI. Protocols for the use of animals in these studies were reviewed and approved by the Institutional Animal Care and Use Committee of the Lankenau Institute for Medical Research in accordance with current US Department of Agriculture, Department of Health and Human Service regulations and standards.

### Cell culture

B16F10-sTAC cells engineered to express SIINFEKL peptide were cultured in DMEM supplemented with 10% fetal bovine serum and 1X Penicillin/Streptomycin. CT26.CL25 cells were cultured in DMEM supplemented with 10% fetal bovine serum, 1X Penicillin/Streptomycin and 0.8 mg/ml of G418 disulfate (Fisher Scientific). CT26.CL25 (American Type Culture Collection, Rockville, MD) is a subclone of CT26 colon carcinoma cells that have been transduced with *Escherichia coli β-gal* gene, which have been shown to be equally as lethal as the parental clone CT26.WT, in normal mice.

### *In vivo* tumor models

Tumor models were established by subcutaneous injections of 5×10^5^ B16F10-sTAC cells in C57Bl6 mice or 5×10^5^ CT26.CL25 cells in Balb/c mice. Mice were monitored twice a week for tumor growth. Treatment with 0.25% (w/v) DFMO in the drinking water and the Trimer PTI (3 mg/kg daily by intraperitoneal injection) was initiated when tumors were palpable (50-100 mm^3^). For adoptive transfers, spleens were excised and pressed through a nylon filter to obtain a single cell suspension. The resulting suspension was incubated with red cell lysis buffer for 5 minutes to lyse red blood cells. Gr1^+^ cells were stained with a PE conjugated antibody and then isolated using anti-PE microbeads (Miltenyi Biotec) as per the manufacturer's instructions. Gr1^+^ positive cells were injected into the retro-orbital cavity. Tumor growth was assessed morphometrically using calipers, and tumor volumes were calculated using the formula V (mm^3^) = π/6 x A x B^2^ (A is the larger diameter and B is the smaller diameter).

### Antigen-specific T-cell response detection by IFN-γ ELISpot

Upon sacrifice, splenocytes from B16F10-sTAC tumor bearing mice and tumor cell suspensions from CT26.CL25 tumor bearing mice were analyzed for IFN-γ producing cells by enzyme-linked immunosorbent spot (ELISpot) assay. Multiscreen filtration plates (Millipore) were coated with 0.5 μg/mL of purified anti-mouse IFN-γ capture antibody (Biolegend) overnight at 4 °C. Single-cell suspensions of splenocytes or tumors were plated at 1×10^6^ and 5×10^5^ per well respectively. For ELISpot assays with splenocytes from B16F10-sTAC tumor bearing mice, cells were stimulated with the SIINFEKL peptide (Anaspec) at 20 μg/mL. Cells from CT26.CL25 tumors were treated with a known H-2D-restricted β-galactosidase peptide (TPHPARIGL; ChemPep). After 16 hours of stimulation at 37 °C, the cells were removed by washing and spots were developed with a biotinylated anti-IFN-γ detection antibody and streptavidin-horseradish peroxidase conjugate followed by NITRO-blue tetrazolium chloride and 5-bromo-4-chloro-3ʹ-indoylphosphate p-toluidine salt substrate (Sigma). Spot numbers were counted, and data were reported as IFN-γ-spot forming cells per 10^6^ cells.

### Immunohistochemistry

Mouse tumor tissues were fixed in 4% paraformaldehyde in PBS overnight and embedded in paraffin. Sections were deparaffinized, hydrated, and then heated in 0.01 mol/L sodium citrate buffer (pH 6.0) in a steamer for 8 minutes. Sections were incubated with the primary antibody (rat monoclonal anti-mouse F4/80 [BioRad MCA497GA]) for 2 hr at room temperature followed by biotinylated secondary and then an avidin HRP complex (Vectastain Elite ABC KIT, Vector Laboratories, INC). Immunoreactive cells were localized by incubating the sections with diaminobenzidine and peroxide and then counterstaining with hematoxylin. Pictures were obtained using a Zeiss Axiophot microscope (Carl Zeiss Inc.) with a digital color camera and corresponding software.

### Cytokine analysis

B16F10-sTAC tumors were flash frozen in liquid nitrogen, ground and homogenized in PBS containing 0.5 μM dithiothreitol, 0.5 μM Pefabloc and 8 μg/mL leupeptin. Samples were analyzed for monocyte chemoattractant protein-1 (MCP-1), interleukin-6 (IL-6). IL-10, tumor necrosis factor alpha (TNF-α) and interferon gamma (IFN-γ) using the Mouse Inflammation Cytometric Bead Array reagents (BD Biosciences, San Jose, CA) and flow cytometry as per the manufacturer's protocol. VEGF cytokine levels were analyzed using the mouse VEGF Quantikine ELISA (R&D systems) as per the manufacturer's instructions.

### Flow cytometry analysis of immune cell infiltrates

Tumor tissue was digested in a 0.3% collagenase/0.1% hyaluronidase solution, pressed through a nylon mesh filter to obtain a single cell suspension and incubated in red cell lysis buffer (0.17 M Tris-HCL, 0.16 M NH_4_Cl) for 5 minutes. Cells were spun down and resuspended in a 40% Percoll solution (GE Healthcare). The tumor cell suspension was then underlaid with an 80% Percoll solution and spun at 325 x g for 23 minutes. Cells at the interface between the 40% and 80% Percoll solutions were removed, washed and prepared for flow cytometric analysis. Equal numbers of viable cells were stained with combinations of the following: CD8a-PECy7, CD4-PE, F4/80-PECy7, CD206-FITC, Gr1-APC, CD11b-PE, CD45-APC-Cy7, Granzyme B-FITC and IFN-γ-APC. Flow-cytometric data were acquired on a BD FACSCanto II cytometer and analyzed using FACSDiva software (BD Biosciences).

### Statistical analysis

All *in vitro* experiments were performed at least in triplicate, and data were compiled from two to three separate experiments. Analyses were done using a 1-way ANOVA with a Tukey test for statistical significance or a Students t-test. *In vivo* studies were carried out using multiple animals (n = 5-10 per treatment group). Tumor growth curves were analyzed with a Generalized Linear model with fixed effects of treatment and time. Data were examined for the interaction between treatment groups and day of observations, testing whether the slopes of the growth curves (tumor volume vs. day of observation) were significantly different for the control and treatment groups. In all cases, values of p < 0.05 were regarded as being statistically significant.

## SUPPLEMENTARY MATERIALS FIGURE



## Abbreviations

DFMO: α-Difluoromethylornithine
MDSC: myeloid derived suppressor cells
ODC: ornithine decarboxylase
PBT: polyamine blockade therapy
PTI: polyamine transport inhibitor
PTS: polyamine transport system
