# Supplementary material for: A novel polyamine blockade therapy activates an anti-tumor immune response
Source: Oncotarget. 2017 Aug 24;8(48):84140–52. doi: 10.18632/oncotarget.20493 (PMC5663583; doi:10.18632/oncotarget.20493)
Supplement: Supplementary file 1 [file oncotarget-08-84140-s001.pdf]

A novel polyamine blockade therapy activates an anti-tumor immune response

SUPPLEMENTARY MATERIALS

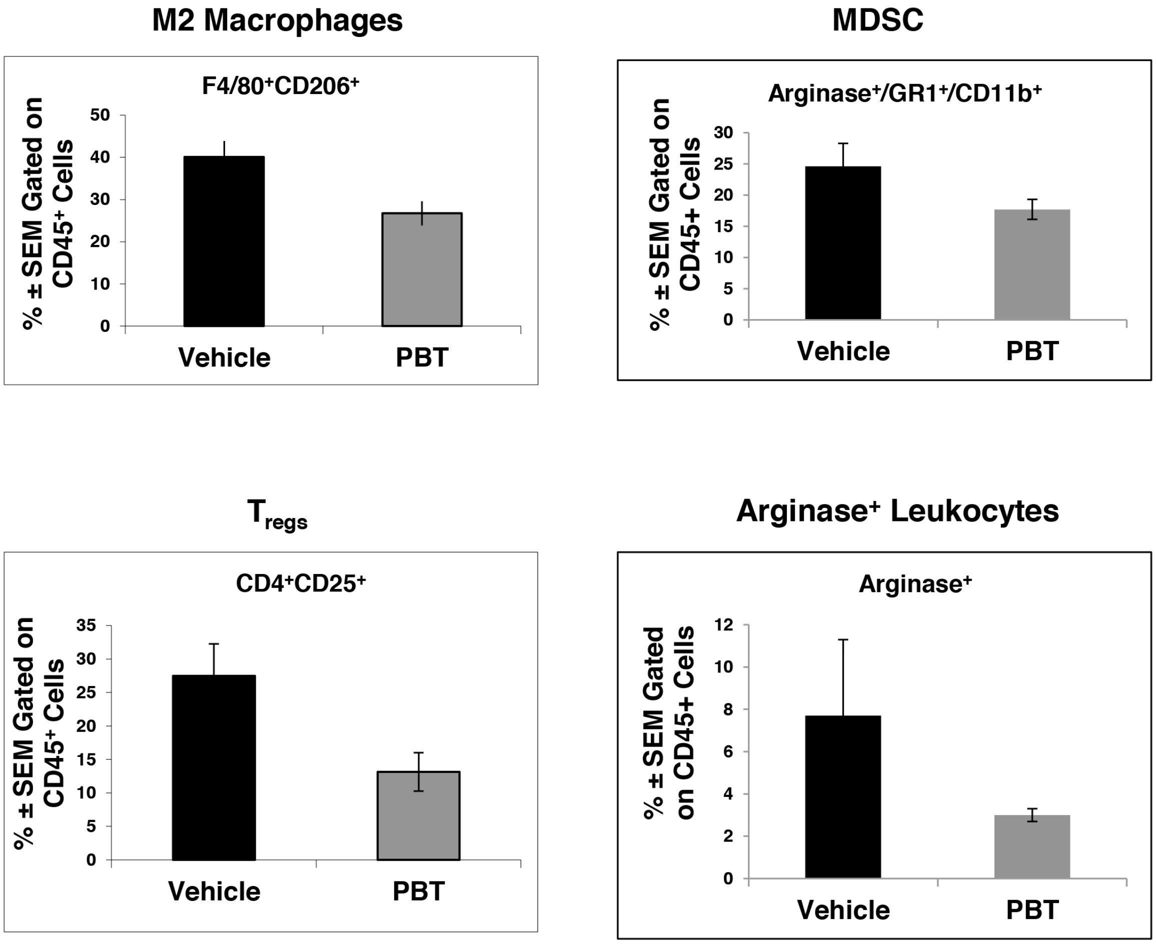

Supplementary Figure 1: DFMO and Trimer PTI co-treatment reduces the immunosuppressive and pro-tumorigenic cells populations in the spleen. Upon sacrifice, spleens were excised from CT26.CL25-tumor bearing mice and processed for analysis by flow cytometry. $CD45^+$ tumor cells were analyzed for the percentage of the indicated cell types. n = 5-10 mice per group. * = $p \leq 0.05$ and # = $p \leq 0.01$ compared to indicated group.
